# Hurdles for adopting mobile learning devices at the outset of clinical courses

**DOI:** 10.1186/s12909-021-03008-9

**Published:** 2021-11-29

**Authors:** Daniel Folger, Jussi Merenmies, Lena Sjöberg, Eeva Pyörälä

**Affiliations:** 1 Clinicum, University of Helsinki, P.O. Box 63, 00014 Helsinki, Finland; 2 Centre for University Teaching and Learning, University of Helsinki, P.O. Box 21, 00140 Helsinki, Finland

**Keywords:** Mobile, Technology, Clinical, Pregraduate, iPad

## Abstract

**Background:**

Mobile devices provide medical students with easy access to medical information and educational resources. Since 2013, we have followed the study use of iPads among medical students. In 2016, we observed a notable drop in the mobile device usage in the first cohort of medical students entering their clinical courses.

**Methods:**

The aim of the study was to identify the hurdles for adopting mobile devices at the beginning of the clinical courses. We examined how students evaluated their own and the clinical teachers’ ability to use the iPad, how the study assignments fit into digital learning, and how students used the mobile device with patients. The data were collected with online surveys among three consecutive student cohorts and the distributions of closed-ended questions analyzed.

**Results:**

Response rates ranged from 67.5 to 90.8%. Students evaluated their own ability to use the iPad as good or excellent and teachers’ skills as relatively poor and wanted more digitally tailored assignments. They reported negative attitudes towards mobile device use in the clinical setting and were hesitant to use them in patient contact. Teachers seldom communicated suitable quality medical applications to students.

**Conclusions:**

Clinical teachers need support and training to implement a learning environment and assignments appropriate for mobile devices. Both students and teachers were concerned about using these devices with patients. To achieve the full potential of digitalisation in clinical courses, their use should be developed collectively with students.

**Supplementary Information:**

The online version contains supplementary material available at 10.1186/s12909-021-03008-9.

## Background

The tablets and smartphones of today provide learners with easy access to massive amounts of medical information and educational resources [1–3]. With the quantity of medical knowledge increasing, no one doctor nor medical student can be expected to remember everything, a problem that is in part solved by mobile technology [2, 4]. As future doctors, today’s medical students are the agents of change, enabling the integration of advantages of mobile devices into clinical patient care [5, 6]. Given the abundance of mobile devices [1, 4] and emerging health technology [5, 7], and further also the integration between user and technology [8], it is imperative to investigate the benefits achieved with mobile technology also in medical education and practice [1–3].

Studies providing an insight into the entry of mobile technology-savvy medical students into the clinical setting are rapidly evolving [3–6]. Overall, mobile device usage in educational contexts has a history of about a decade, and in accordance with the novelty of the phenomenon the studies on device usage have only recently accumulated [1, 9] despite full iPad curriculums having existed since 2011 [3]. Studies so far have found that students and junior doctors use mobile devices for searching information, time management, retrieving information before treating patients, reporting to senior colleagues, and most importantly for backing up their clinical reasoning and decision making [2, 10], but more seldom in direct patient contact [6].

Studies focusing on mobile device usage in the clinical context have repeatedly raised the topic of hesitance in using the device with patients and have raised a serious concern of patients’ reactions to mobile device usage, fears being that the students’ devices would have a deteriorating effect on communication with the patient and could be interpreted as a sign of uncertainty and unprofessional behaviour [2, 7]. However, a few studies among patients have suggested that they were not bothered by the use of mobile technology but were pleased that physicians used devices bedside to assist them in diagnostic decision-making [6, 11].

The uncertainty of patients’ reactions has not been the only major obstacle in adopting mobile technology in the clinical context. Students’ have also faced social barriers in the form of their device usage and online information seeking being construed as unprofessional behaviour and misinterpreted as personal use of social media by senior doctors and other healthcare professionals [12–14].

Poor Internet access in teaching hospitals has also been reported as one of the major barriers to the use of mobile devices. Studies have reported that students have had no Wi-Fi access in the hospital or there have been dead spots in the network coverage. This has resulted in an inability to access the Internet and digital materials and consequently frustration among students [11, 15]. Furthermore, many hospitals have regulated the use of personal mobile devices through formal policies, thereby hindering the open use of devices [2]. Previous studies have suggested that medical students and junior doctors seek reliable medical mobile resources [15, 16] and would like senior colleagues to share with them quality online resources in their field. Medical students’ learning in the clinical context is also found to be best supported when the clinical teachers command effective teaching methods and the use of modern learning technology [17].

From 2013 to 2018, one Finnish Faculty of Medicine delivered iPad tablet computers to first-year medical and dental students for their personal study use. An action research project has followed medical and dental students’ use of iPads since then, collecting data on all new student cohorts and followed their mobile device use in both their pre-clinical and clinical courses [18]. Therefore, an opportunity presented itself to explore the student cohorts’ device use both vertically, following the development of a cohort during their study years [19], as well as horizontally comparing the device use of different student cohorts at certain stages of their studies. The questionnaire contained partly the same questions for students of all study years, but new items were included to match the clinical learning environment. Our search of the literature has not discovered a similar study.

We found early on that both students and teachers needed support to learn how to use these devices for educational purposes, which were offered to them in various forms such as iPad training, pop up support, and e-guides [18]. Most of the students were persistent in learning to use the new device as a learning tool [18]. We observed [19] a peak in mobile learning in the biomedical sciences courses in the first and second study years in which all the learning materials were delivered in the digital format before class and the electronic learning environment was well designed. Digital note taking was along with online information seeking the most important use of mobile devices in students’ studies [18]. One of the key findings of the project to date has been that when the first medical cohort began clinical trials in spring 2016, their iPad use declined significantly [18].

This study is explorative and based on the principles of action research. The research strategy was designed to examine changing practices and address educational challenges faced by medical students when embarking on clinical courses with mobile devices [20]. We sought to identify challenges and impediments to be overcome in the clinical setting in order for us to discover feasible ways of using mobile devices and ways to make the most use of the potential of the new technology in the hands of future healthcare providers. We sought to answer the following questions: (1) How did the students assess their own and their clinical teachers’ ability to use iPads in learning and teaching? (2) How did the clinical pre-class and in-class assignments support the students’ use of the new technology? (3) How did students use the mobile device with patients?

## Methods

To explore hurdles in the adoption of iPads at the outset of the clinical courses, we used an online survey designed for this study and collected data from the three consecutive student cohorts entering the clinical courses and focused on the closed-ended questions concerning in-class digital teaching and learning, and the use of iPads in teaching involving patients.

### Context of the study

The context of this study was a medical degree programme at a Faculty of Medicine in Finland which admits new students every autumn. The first 2 years focus mostly on biomedical sciences. Students learn these topics through problem-based tutorials and lectures. The clinical portion of the medical degree program includes lectures, small group -teaching, skills lab exercises, bedside teaching, and hands-on clinical learning on hospital wards and outpatient clinics. The medical programme rewards the title of Licentiate of medicine, qualifying graduates to practice medicine in Finland.

### Participants

The three cohorts studied consist of iPad-equipped 3rd year medical students which commenced their medical studies in 2013, 2014 and 2015, and further started their clinical courses in the spring of 2016, the autumn of 2016, and the autumn of 2017 respectively. Before the term of 2016–2017 the clinical phase of the degree programme traditionally commenced during the spring term of year 3 but was then shifted to start at the beginning of year 3 due to a revision of curriculum. This study analysed responses given by the cohorts at the end of their respective first semester of clinical courses.

Basic information on cohorts, including the number of students per cohort, response percentages and sex of respondents were also gathered and are presented in the Results.

### Data collection and analysis

The data were collected using online surveys (E-lomake©) designed for the research project. The survey included closed-ended multiple choice and 5-point Likert scale questions, and open-ended questions on the study use of mobile devices. No validated questionnaires were available upon review of the literature. The questionnaires were developed from themes arising from the literature and the teaching and learning practices in our unit, and specific items related to the use of mobile technology for clinical courses were added to the survey. The questionnaire was originally in Finnish. The translation of the questionnaire is provided in the Supplement. This study analysed responses given by the cohorts at the end of their first clinical semesters. Several reminders per survey were sent to maximize the participation rates.

Duplicate responses were checked and the latter removed in the raw data. Answers stored through the electronic questionnaire service were exported to Microsoft® Excel and the principal investigator (EP) anonymised the data by deleting the student identifiers from the data. The closed-ended questions were analysed using Microsoft® Excel for Mac version 16.22 by calculating the distributions of the answers to the statements on the five-point Likert scale and comparing the results between the three cohorts.

## Results

The response rates for the surveys were satisfactory, 90.8% for the first cohort, 70.8% for the second cohort and 67.5% for the third cohort (Table 1). The response rates of female and male students correspond to their portion of the whole student cohort. The age of the respondents per cohort ranged between 22 and 39, 21–41 and 22–50. The first two cohorts were comprised of 120 medical students each, while the 2015 intake was 150.

**Table 1:** The response rates, gender and age of respondents of the three third year medical student cohorts in the years 2016, 2017 and 2018

	Total	Female	Male	Ages 18–21	Ages 22–25	Ages 26–29	Ages 30+
1st cohort in 2016 (*N* = 109)	90.8%	57.9%	42.1%	0	80	18	11
2nd cohort in 2017 (*N* = 85)	70.8%	54.1%	45.9%	1	57	17	10
3rd cohort in 2018 (*N* = 81)	67.5%	56.8%	43.2%	0	55	13	13

When the 2016 cohort started their studies in 2013, 80.7% of the respondents had a smartphone and 19.2% had in addition to the iPad another tablet computer whilst 77.1% had a laptop (Table 2). The adoption of the smartphone is obvious in the subsequent cohorts with almost all students owning one in the 2018 cohort, and the share of students entering studies with only a smartphone almost tripling over 3 years. Tablet computers also became increasingly common, one third of newly accepted students owning one by 2015. Meanwhile the incidence of laptops amongst new students remained at a fairly stable level.

**Table 2:** Students’ own devices at the beginning of their studies in 2013 through 2015

	Smart phone	Tablet computer	Laptop computer	Only smartphone in use
1st cohort in 2013	80.7%	19.2%	77.1%	8,8%
2nd cohort in 2014	92.1%	23.8%	80.2%	13,7%
3rd cohort in 2015	99.1%	34.3%	75.9%	23,1%

### Students’ and teachers’ ability to use iPads in learning and teaching

**Fig. 1:**
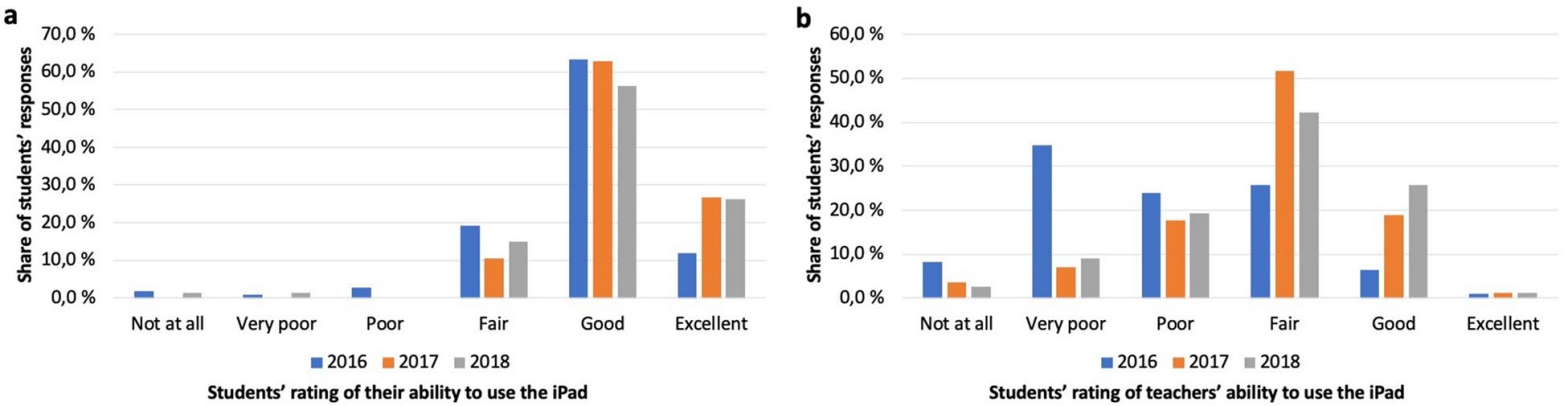
Ability to use the iPad (**a**) Students’ own ability to use the iPad in studying; **b** Teachers’ ability to use the iPad in teaching

Students were asked to rate their own ability to use the mobile device for studying and subsequently rate their clinical teachers’ ability to use the iPad in teaching. Students generally rated their own ability of using the iPad in studies as good, the incidence of reported excellent usage doubling for the latter two cohorts. Reports of lacking or poor ability to use the iPad in studies was almost non-existent. The 2016 cohort deemed only 7.3% of teachers to use iPads for teaching in a good or excellent fashion, and 43.1% to do so inadequately or not to be able to use them at all. Subsequent cohorts reported increasing rates of good usage of the iPad by teachers, also fair usage seeing a notable increase. The reported rate of very poor usage decreased markedly after the initial cohort (Fig. 1).

### Distribution and format of study materials

**Fig. 2:**
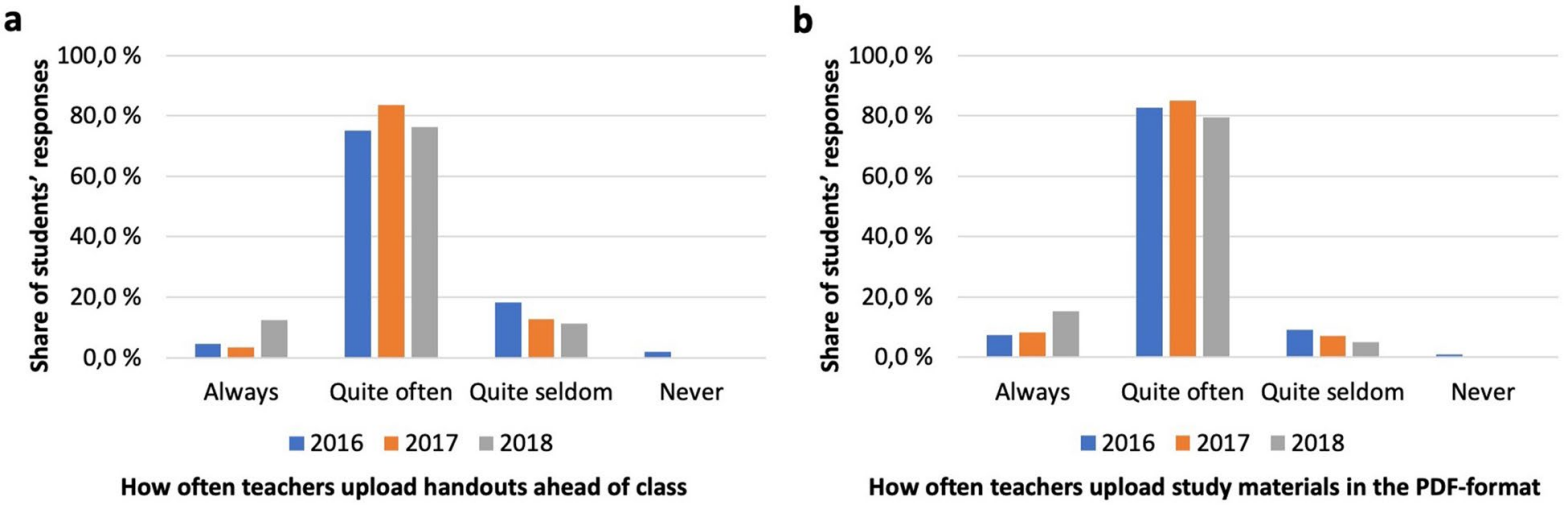
Uploading of study materials (**a**) Teachers’ uploading of handouts in e-learning environments before class; **b** Teachers’ uploading of study materials in the PDF format

To effectively use mobile devices for note taking, students needed to download teachers’ handouts in time before class in a suitable format (in general PDF). We asked the students about utilization of electronic study materials. Seventy-five percent of the 2016-cohort reported teachers to upload handouts before class quite often, similar responses being given by the latter two cohorts (Fig. 2a). 18% of the 2016 cohort thought teachers to do this quite seldom with a steady decline in latter cohorts. The number of students reporting study materials always to be uploaded ahead of class increased notably in the last cohort, the absolute number however still being low.

The majority of the 2016 cohort reported the PDF format to be used quite often, subsequent cohorts reporting similar rates (Fig. 2b). Only a fraction of the 2016 cohort thought teachers to do this quite seldom, the incidence decreasing with subsequent cohorts. A noticeable finding is that 15% of the 2018 cohort thought teachers to do so always, an increase from the previous 2 years’ figures.

### Use of mobile device-compatible pre-assignments

**Fig. 3:**
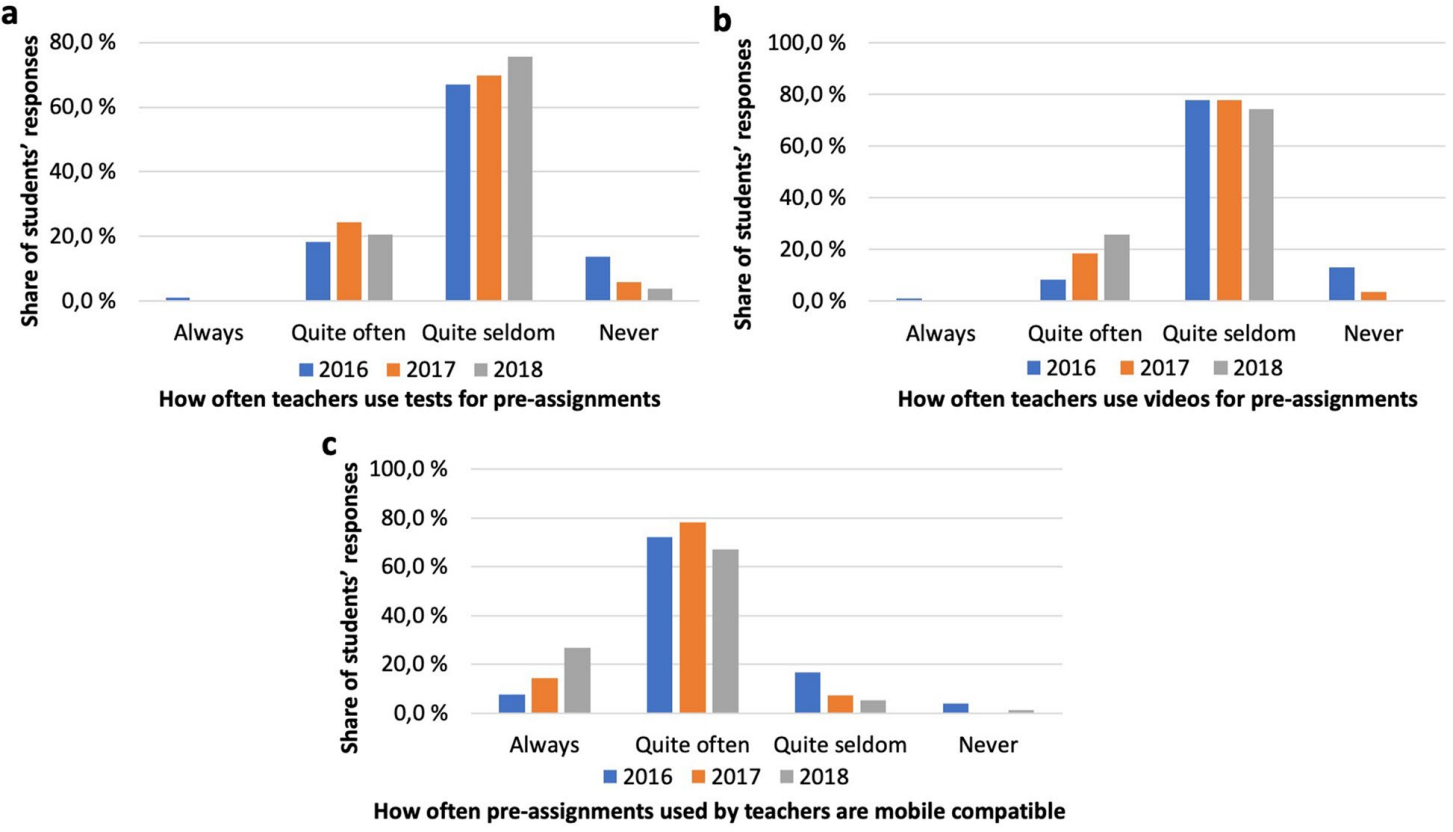
Use of pre-assignments (**a**) Teachers’ use of tests for pre-assignments; **b** Teachers’ use of videos for pre-assignments; **c** Mobile compatibility of pre-assignments used by teachers

We asked the students to evaluate how the pre-assignments were implemented in their courses. A majority (67%) of the 2016-cohort reported teachers to use tests for pre-assignments quite seldom, the number rising with the following cohorts (Fig. 3a).

Videos were used for pre-assignments quite often according to only a fraction of the 2016 cohort, reported rates rising with the subsequent cohorts (Fig. 3b). Over three quarters of the 2016 cohort thought videos to be used quite seldom with similar response rates in the subsequent cohorts.

A steadily increasing number of students reported pre-assignments used as being mobile device compatible, and conversely a decreasing number thought them to be so quite seldom (Fig. 3c).

### Use of in-class triggers and applications

**Fig. 4:**
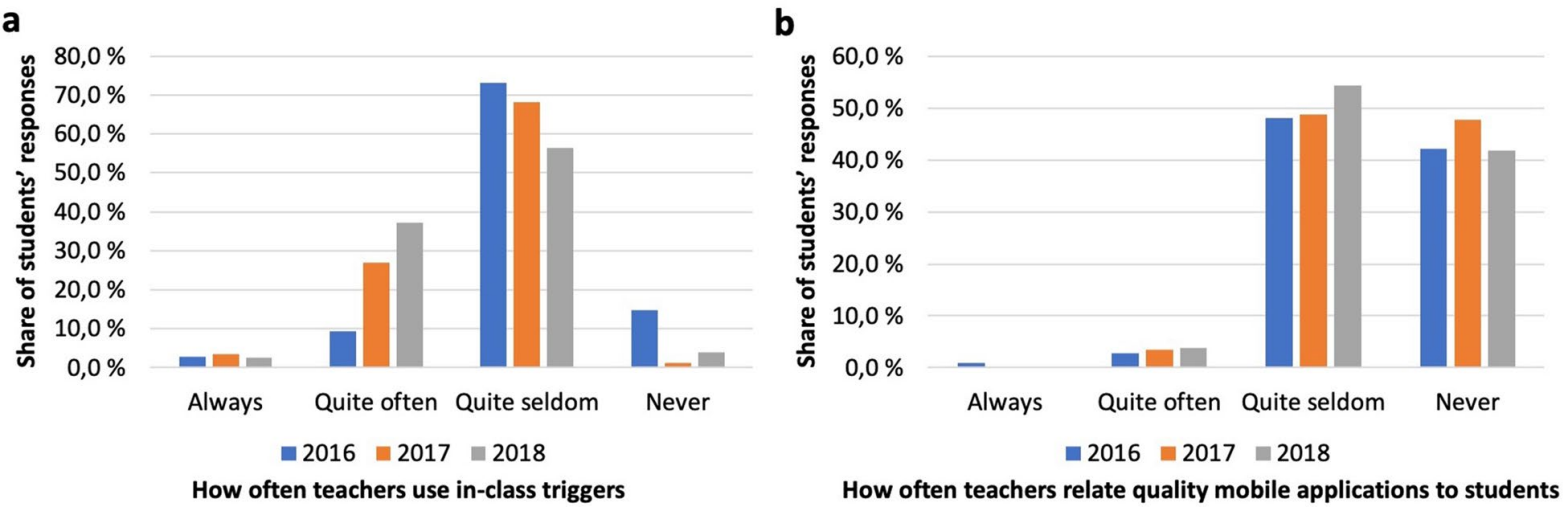
In-class triggers and apps (**a**) Teachers’ use of in-class triggers (voting, tests and tasks); **b** Teachers relating of quality mobile device applications to students

Only a mere 12.6% of the 2016 cohort reported teachers to always or quite often use in-class triggers and applications for enhancing learning, subsequent cohorts however reporting markedly increasing rates (Fig. 4a). Respectively, a clear majority of the first cohort thought teachers did so quite seldom, subsequent cohorts reporting a continuous increase.

A considerable number of resources and applications designed for medical education are available in application stores. The survey showed that teachers seldom or never told students about apps relating to their clinical field, results staying similar throughout all studied cohorts (Fig. 4b).

### Students' use of iPads in studies

**Fig. 5:**
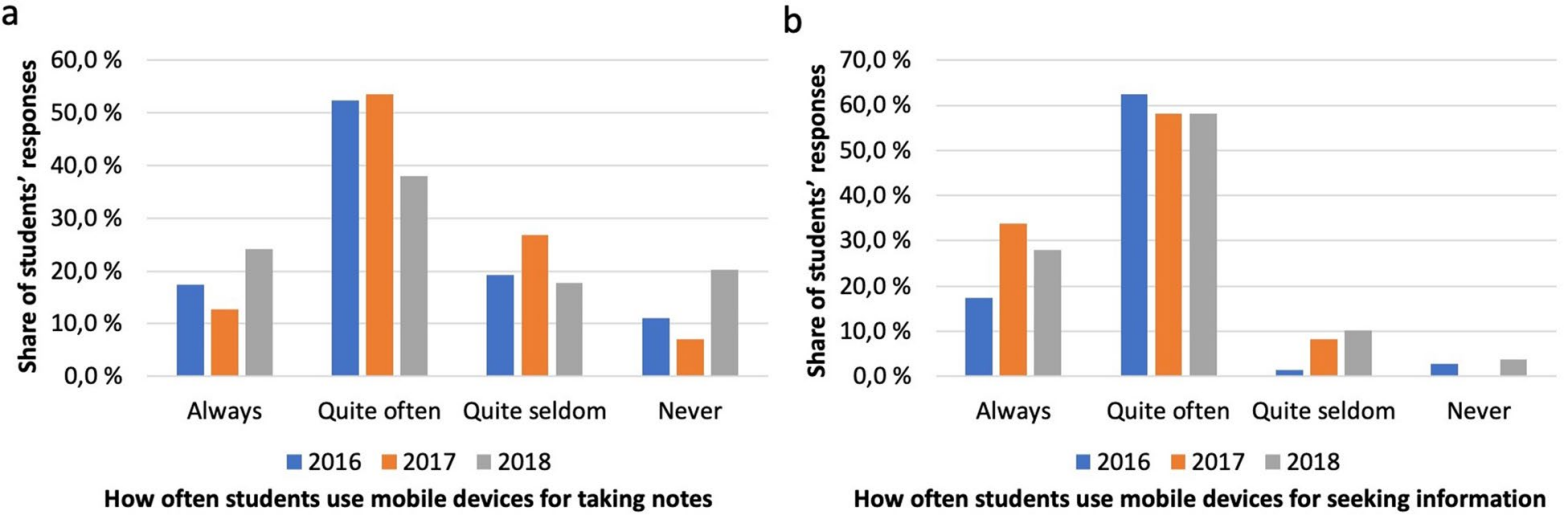
Note taking and information seeking (**a**) Students’ use of mobile devices for taking notes; **b** Students’ use of mobile devices for seeking information

Students used iPads for note taking (Fig. 5a) and information seeking (Fig. 5b). The incidence of students reporting always using mobile devices for seeking information saw almost a doubling after the first cohort, whilst the majority reporting doing so quite often remained at a constant level (Fig. 5b). Those reporting using mobile devices for seeking information quite seldom saw a steady increase whilst remaining only a fraction of the respondents.

### Students’ use of mobile devices with patients, and the remedies they suggested for improvement

**Fig. 6:**
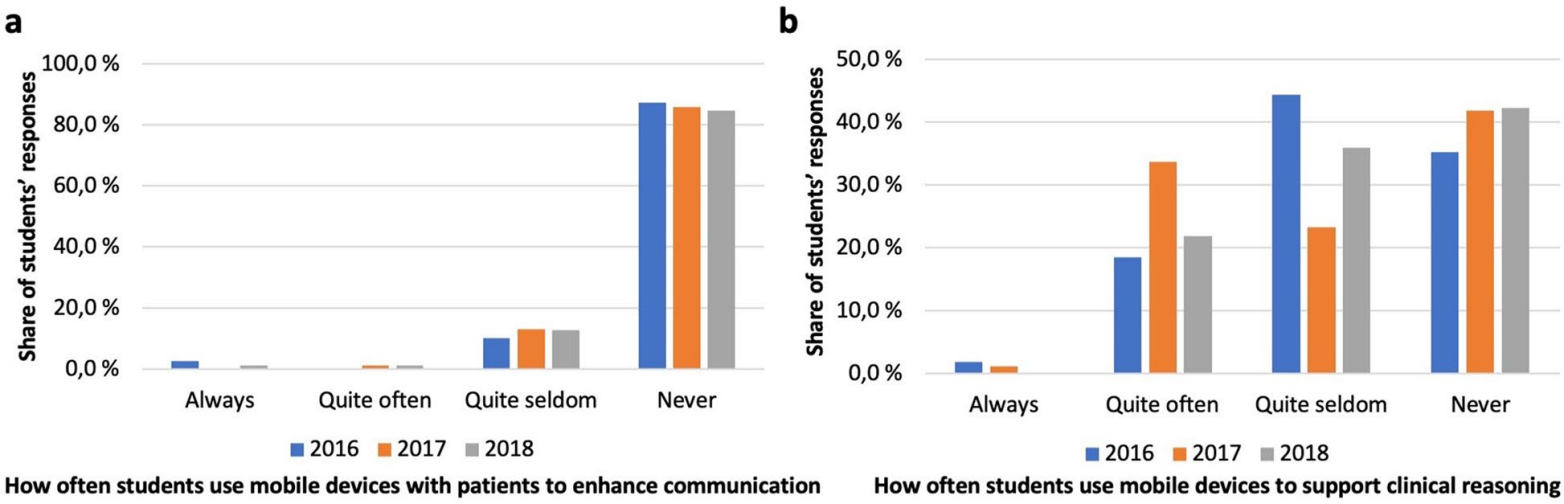
Use of mobile devices in the clinical setting (**a**) Students’ use of mobile devices with patients to enhance communication: **b** Students’ use of mobile devices to support clinical reasoning

Use of mobile devices with patients for enhancing communication was sparse and on the border of non-existent with only a tenth of the 2016 cohort reporting using devices quite seldom and a clear majority reporting never using mobile devices for this purpose (Fig. 6a). Subsequent cohorts reported very similar numbers.

A mere fraction of students reported always using mobile devices for supporting clinical reasoning. 19% of the 2016 cohort students reported using a mobile device quite often for supporting clinical reasoning, latter cohorts reporting varying rates (Fig. 6b). Almost half of the 2016 cohort reported seldom usage with latter cohorts’ rates varying. Roughly a third of the 2016 cohort and later slightly higher numbers of students reported never using a mobile device for clinical reasoning.

### Use of patient records on mobile devices

**Fig. 7:**
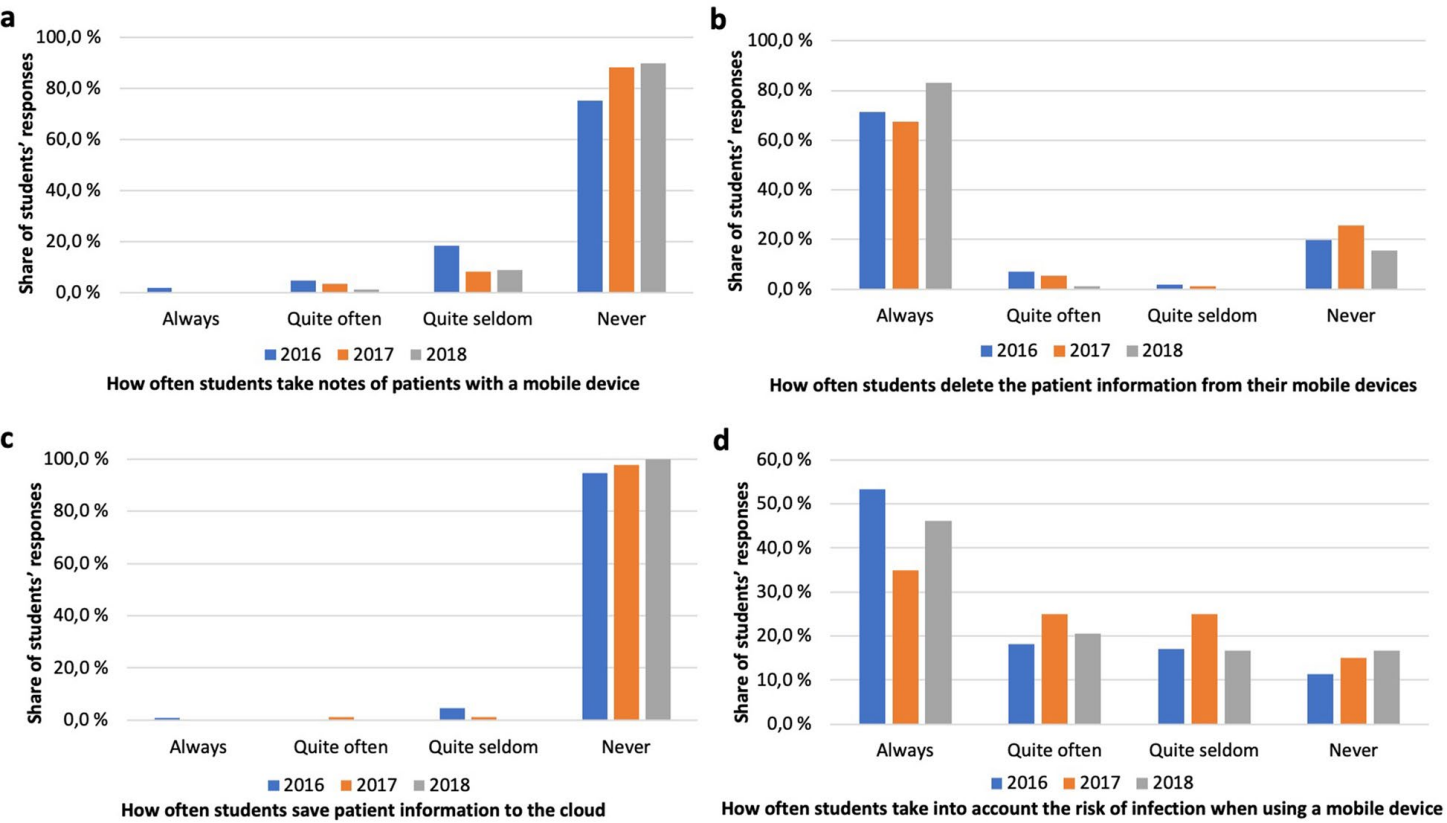
Patient information and risk of infection (**a**) Students take notes of patients with a mobile device; **b** Students delete the patient information from their mobile devices; **c** Students save patient information to the cloud; **d** Students take into account the risk of infection when using a mobile device

The privacy of patients and the security of their information is pivotal in healthcare. The majority of students through each cohort reported never using mobile devices for taking notes on patient information (Fig. 7a). The majority also reported always deleting any patient information stored on the device (Fig. 7b), with around a fifth of students reporting never to do so. The decision not to store patient information on cloud services was almost unanimous with close to all respondents reporting never to do so (Fig. 7c).

The risk for transmission of infection requires attention when adopting mobile devices in healthcare. Students reported taking into account the risk of infection more often than not (Fig. 7d), 71% (2016) reporting to do so always or quite often, with corresponding figures for the subsequent cohorts decreasing slightly but staying above 60%.

## Discussion

The aim of this study was to explore hurdles in the adoption of iPads in the clinical phase of the medical degree, as well as to examine differences in hurdles faced by the three consecutive years of iPad-cohorts entering clinical education. This was to get an idea of how well the clinical environment was receiving the tablet computers given their value as aids in learning clinical medicine. The response rate of the first cohort in 2016 was as high as 90% which is in line with the novelty of the use of iPads in the clinical setting. Decrease in the response rates with subsequent cohorts is likely due to questionnaire fatigue. The use of mobile phones, tablets, and laptops by new students at the beginning of their degree studies largely mirror overall adoption of mobile technology in the general population.

The same type of research on the use of new technology by successive medical student cohorts in the early stages of clinical courses had not been conducted before. However, our results were consistent with previous research findings [1, 3, 14]. Both previous research literature and our own previous research on the first cohort of medical students reported challenges in incorporating devices into clinical settings [12, 16, 18, 19]. For students, this was problematic because they were accustomed and proficient in using mobile devices in the biomedical science courses in their first two study years [18], and it was in the clinical setting that the importance of online information sources increased as they participated in patient care and needed immediate access to e.g. clinical databases. Eventually, they ended up using their personal smartphones for this purpose. Resistance did not end completely with the first cohort, but the use of mobile devices for information retrieval, in turn, increased significantly among students in the second and third cohorts. This may be due to the general increase in the use of mobile devices, good practices passed on by older students to their younger colleagues, reporting preliminary results to teachers on the clinical phase challenges of the iPad project [19], and tailored support for clinical teachers [18].

As reported in previous studies [12, 13], students experienced ambivalence in mobile device use with patients. We observed that any mobile device use in direct patient contact raised serious concerns among students. They were also worried that using the device might deteriorate their communication with the patients [2, 7, 12, 13]. In addition, they feared that senior physicians and other health care professionals would construe their behaviour as unprofessional or misinterpret their online information seeking for personal communication on social media [12–14]. The evident lack of role models for the use of mobile devices in the clinical setting speaks to the need for the support of faculty development pertaining to the use of these devices. The few studies exploring the patients’ own perceptions of bedside usage of mobile devices reported positive attitudes among patients [6, 10, 11]. We have not yet collected data from a patient perspective, but we believe that using the new technology together with patients would be an important target for future research. In addition, the use of mobile devices in a clinical setting requires open guidelines and codes of conduct [7, 13].

Previous studies reported that poor Wi-Fi access and therewith problems with accessing the Internet and digital learning materials in the clinic were major barriers to benefiting from mobile devices in the clinical context [11, 15]. In our own earlier study, students who were accustomed to receiving learning materials in biomedical courses on time and in a format suitable for mobile devices complained that in clinical courses, materials were delivered too late and in an inappropriate format for taking notes [19]. Because clinical teachers rarely used digital pre-assignments or online assignments in the courses, several students eventually left the iPad at home. Comparing the results between the three cohorts entering the clinic, there was a slight positive trend towards more active use of the digital learning environment and better reception of the mobile-savvy students in the clinical context over time.

We found a significant discrepancy between students’ own reported ability to use iPads for study and their teachers’ ability to apply these devices to teaching. Both improved with the following cohorts. A comparison of the three cohorts showed that the ability of teachers to apply the digital environment effectively and accept students’ mobile devices improved relatively slowly. This suggests that it is important that teachers receive adequate training and support in the use of new technologies. On the other hand, it is important to support teachers in gaining practical knowledge of the format in which the learning materials should be distributed electronically, evident by the lower incidence of format-appropriate materials used compared to preclinical courses [19]. The knowledge of how to create and implement digital assignments as part of the course is central for executing a mobile friendly curriculum.

An important finding of this study was the fact that practically no students seemed to use the iPad for backing up clinical reasoning. This despite a majority reporting using it for seeking information. The results may be in part explained by difficulties in interpreting the questionnaire statements. Some students might also be unfamiliar with the concept of clinical reasoning so early in their clinical courses.

Another noteworthy finding lies in the mobile devices use for taking notes on patient information. Whilst students almost unanimously reported never taking notes on patient information with their mobile devices, ca 20% of students from each cohort reported never deleting patient information from their device. The discrepancy may indicate that more students used their iPad for taking some notes on patient information than cared to admit it in the questionnaire, or some students simply felt they would not need to delete patient information if they took notes of them using their iPad. The possibility of difficulty interpreting the question remains. While working in healthcare units, students are only allowed to write patient record information under supervision, but they participate in bedside instruction and examine patients, and are allowed to fill out paper forms when interviewing and examining a patient. Despite the potential of mobile devices for quickly storing patient information e.g. through photography and note taking or copying of information, both legal and ethical boundaries limit patient record use on any device not sanctioned by hospital administration.

Mobile devices are in themselves powerful devices, even using only a web browser. However, the mobile revolution has also been based on the development of innumerable medical applications. In line with previous research [15, 16], our study showed that teachers provided students with very little information about high-quality medical applications [19]. These results remained similar in all three cohorts. The novelty of mobile devices in clinical teaching may be one explanation for this, but over time the question arises as to why so few clinical teachers recommended good medical applications to students or used applications as part of their clinical teaching [15, 16, 19].

### Strengths and weaknesses of the study

The strength of this study is found in part in the duration of the iPad research project, having started in 2013, and data having been gathered on all subsequent student cohorts until 2020. No similar study has yet been found to track cohorts of medical students using their mobile devices throughout the degree. Furthermore, a total of three cohorts were analysed for this study, which provided results on the use of mobile devices in clinical courses from their introduction to routine use. The response rates of the study were satisfactory. With the lead author being a student of one of the studied cohorts also unique insight into the phenomenon studied was utilized. The research project has actively collaborated with both students and faculty, yielding benefits from the iterative process. This study delivers on previously identified interesting results that have been reported in international conferences and published in peer-reviewed articles [18, 19].

There are also limitations in this study. We did not find a validated questionnaire in the research literature on the use of mobile devices in medical education, so we designed the survey ourselves. In addition, one limitation was that it was performed in a single medical education unit. However, as no such study had been published, we thought that a detailed study of students’ perceptions of barriers to mobile device adoption in a clinical setting would provide useful information for units incorporating new technology into their teaching. The Covid-19 pandemic has also increased interest in and utilisation of online learning in all medical education units [21].

### Future directions

A large amount of both quantitative as well as qualitative data has been collected as part of the iPad research project from 2013 through 2020. The next step is to analyse the students’ answers to the open-ended question using qualitative content analysis. In addition, it is interesting to study how these student cohorts responded to mobile learning surveys in later courses. In this study, the quantitative items of the questionnaires were used, in which students responded to the statements on the Likert scale. In our future report, we will focus on the qualitative analysis of open-ended responses and will better be able to answer questions regarding e.g. the affordances of iPad use in the clinical phase of a medical degree.

Further topics to look into will be patients’ perception of the use of mobile devices in clinical encounters, particularly by involving patients in the development of the use of digital devices and applications in healthcare. The use of social media by students and young doctors as sources of medical information is also an important topic that should be examined. In addition, the change in mobile device use (e.g. utilisation of QR-codes etc. for social distancing, telemedicine developments) after the COVID-19 pandemic would be interesting to study but is beyond the scope of this article as the data for this study were collected before the pandemic. Several studies on the use of mobile devices have been published and the mobile devices usage in the new normal is poised to be an interesting study area [22–25]).

## Conclusion

There were three main findings related to the hurdles for adopting mobile learning devices at the outset of the clinical studies. First, there was a mismatch in students’ and teachers’ ability to apply the mobile device in teaching and learning. Second, the digital learning environment did not support mobile note taking nor other use of novel technology in the students’ first clinical courses. Third, the ambivalence related to the usage of the mobile device in patient contact was twofold. Students themselves hesitated to use the devices with patients and also feared the attitudes of the senior colleagues and other healthcare professionals towards their device use. Taking into account these aspects would benefit the utilization of the full potential of mobile devices in clinical studies, making way for incorporating them and making the most of their benefits in future patient care.

## Supplementary Information

**Additional file 1.** Message to the students

**Additional file 2.** Questionnaire translated into English

## Data Availability

The research data analysed during the current study are available from the corresponding author.
