# Supplementary material for: Hurdles for adopting mobile learning devices at the outset of clinical courses
Source: BMC Med Educ. 2021 Nov 29;21:594. doi: 10.1186/s12909-021-03008-9 (PMC8629605; doi:10.1186/s12909-021-03008-9)
Supplement: Supplementary file 1 — Additional file 1. Message to the students [file 12909_2021_3008_MOESM1_ESM.docx]

**Message to the students and the questionnaire translated into English**

Dear medical/dental student,

The Faculty of Medicine provided you with iPads for personal study use in 2013. The Faculty established an iPad research group for developing the study use of iPads in medical and dental studies in our faculty. The iPad research group invites you to give information about the study use of iPads to improve the study use of these devices.

You are our best experts in the use of mobile devices for studying. We wish you to answer a questionnaire concerning your personal study use of the device in medical/dental studies.

Answering the questionnaire is voluntary. The answers will be treated confidentially and anonymity is guaranteed throughout the analysis and the report of the results.

You can answer the questionnaire by using the following online format. Remember to send your answers by pressing the end command at the end of the questionnaire.

<https://elomake.helsinki.fi/lomakkeet/1234/lomake.html>

We recommend you fill in the online format in a place where the wifi is stable.

Your response is valuable to us!

Eeva Pyörälä

Senior Lecturer in University Pedagogy

Faculty of Medicine

Tel. 050-3491553

e-mail: [eeva.pyorala@helsinki](mailto:eeva.pyorala@helsinki).fi
